# Target Coverage in Wireless Sensor Networks with Probabilistic Sensors

**DOI:** 10.3390/s16091372

**Published:** 2016-08-27

**Authors:** Anxing Shan, Xianghua Xu, Zongmao Cheng

**Affiliations:** 1 School of Computer Science, Hangzhou Dianzi University, Hangzhou 310018, China; 141050025@hdu.edu.cn; 2 School of Science, Hangzhou Dianzi University, Hangzhou 310018, China; zmcheng@hdu.edu.cn

**Keywords:** wireless sensor networks, target coverage, probabilistic sensor

## Abstract

Sensing coverage is a fundamental problem in wireless sensor networks (WSNs), which has attracted considerable attention. Conventional research on this topic focuses on the 0/1 coverage model, which is only a coarse approximation to the practical sensing model. In this paper, we study the target coverage problem, where the objective is to find the least number of sensor nodes in randomly-deployed WSNs based on the probabilistic sensing model. We analyze the joint detection probability of target with multiple sensors. Based on the theoretical analysis of the detection probability, we formulate the minimum *ϵ*-detection coverage problem. We prove that the minimum *ϵ*-detection coverage problem is NP-hard and present an approximation algorithm called the Probabilistic Sensor Coverage Algorithm (PSCA) with provable approximation ratios. To evaluate our design, we analyze the performance of PSCA theoretically and also perform extensive simulations to demonstrate the effectiveness of our proposed algorithm.

## 1. Introduction

With the advancement of embedded sensors and wireless networking technology, WSNs have become one of the most important research areas, which arouses tremendous interest from both the academic and industrial communities. Wireless sensor networks are widely applied in many applications, such as environmental monitoring, forest-fire prevention and air quality monitoring [1,2,3,4]. The coverage problem concerns how well a sensor network is monitored or tracked by sensors, and it is one of the fundamental issues in sensor networks. Three categories of the coverage problem exist in the literature based on the coverage subject [5,6]: target coverage (e.g., [7,8,9,10,11,12,13,14,15,16,17,18]), area coverage (e.g., [19,20,21,22,23,24,25,26,27,28]) and barrier coverage (e.g., [29,30,31,32,33,34]).

This paper focuses on the target coverage problem. Targets are often modeled as a set of discrete space points within the sensor field. These points can be used to represent physical targets or static events. Sensors are typically deployed in two schemes: (1) deterministic deployment, which is planned with fewer sensors, but is more time consuming and labor intensive; and (2) stochastic deployment with low labor cost by vehicles or aircraft [26]. Given the stochastic deployment of a sensor network, the objective of the target coverage problem is to activate a subset of sensor nodes to monitor targets, which are randomly distributed on the 2D plane. Existing solutions to sensor coverage mostly leverage the 0/1 disk model, i.e., in the area coverage problem [26], the basic 0/1 disk model is used to model sensors covering a 3D surface, and in barrier coverage [35], the same model is used to model mobile sensors forming a *k*-barrier.

**Figure 1:**
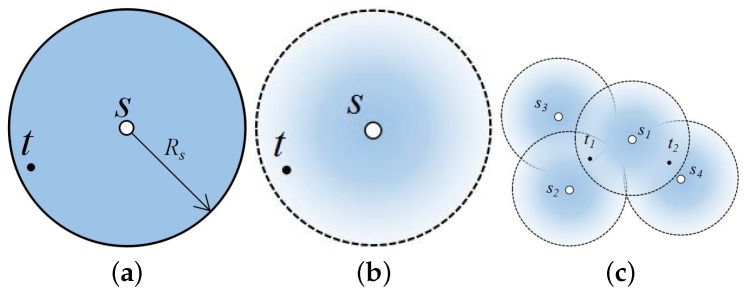
The difference between the idealized 0/1 disk model and the probabilistic sensing model. (**a**) Idealized 0/1 disk model; (**b**) probabilistic sensing model; (**c**) the target $t_{1}$ needs three probabilistic sensors to achieve threshold *ϵ*, while $t_{2}$ needs two.

In the 0/1 disk model, a target within the sensing radius of a node is always assumed detected with a probability of one, while outside this circle, it is assumed not detected. This idealized model is based on unrealistic assumption of perfect coverage in a circular disc. In practical applications, however, the sensing capabilities of sensors are affected by environmental factors in real deployment, especially for acoustic sensors. As shown in Figure 1a for the acoustic sensor, target *t* is assumed covered by sensor *s* based on the 0/1 disk model. However, as the received energy attenuates with distance, the acoustic sensor can hardly detect target *t* when it is close to the edge. In this case, the 0/1 disk model is no longer practical. The probabilistic sensing model, which can characterize the quality of sensor coverage more accurately, has been proposed with the assumption that sensing probability is a decreasing function of the sensing distance [36]. Different from the 0/1 disk model, target *t* in Figure 1b is detected by sensor *s* with a probability between zero and one. A target is considered covered when its detection probability is beyond a threshold. Therefore, the target usually needs two or more sensors for collaborative detection.

Much work has been done to address the target coverage problem; however, few of them consider the probabilistic model. In this paper, we focus on the target coverage problem with omnidirectional probabilistic sensors. We formulate the target coverage problem based on omnidirectional sensors deployed with a probabilistic model. The problem is fundamentally different from the target coverage problem based on the 0/1 disk model, since the coverage requirement ensures that the detection probability of each target is at least *ϵ*. We then aim to find the minimum number of probabilistic sensors to cover all targets with a detection probability greater than a threshold, i.e., at least *ϵ*. As a sensor network may involve tens of hundreds of nodes in a real deployment, the total energy consumption is typically large. It is crucial to only activate as few sensors as possible, especially for the sensors with high energy consumption. Therefore, our objective is to maintain the detection probability of each target as no less than *ϵ* with the least active sensors, i.e., achieve the minimum *ϵ*-detection coverage for all targets.

There exist several challenging issues in finding an effective solution to the above problem. Firstly, since sensors with a probabilistic model usually have no explicit detection boundaries, the detection probability is calculated accurately only by taking all active sensors into consideration, which has high computational complexity. Since the detection probability decreases with respect to the distance between target and sensor, the further away sensors have little sensing impact on the targets, and hence, they can be typically ignored. Therefore, it is meaningful to set a distance threshold as the detection boundary of the sensor. The detection probability is assumed as zero when the target is outside the detection boundary. However, if the detection boundary is set too large, it may end up with much unnecessary computation, which should be avoided. On the other hand, if the detection boundary is too small, a sensor’s detection range has a limited scope. In this case, we may not achieve the detection probability threshold *ϵ*. Due to different detection probability thresholds, it is a non-trivial task to determine the proper distance boundary. Secondly, we show that the minimum *ϵ*-detection coverage problem is NP-hard. Therefore, it is challenging to determine the least active sensors in polynomial time. Finally, due to the uncertainty of probability detection, the target coverage problem will become more complex and is far more challenging than the 0/1 target coverage problem. Each target requires a different number of probabilistic sensors when the probabilistic deployment model is used, while only one sensor is needed for every target when the 0/1 disk model is used. As shown in Figure 1c, because sensors $s_{1}$, $s_{2}$ and $s_{3}$ are far away from target $t_{1}$, we must activate all of them to detect $t_{1}$ with a probability beyond *ϵ*. However, for sensor $s_{4}$, which is close to target $t_{2}$, only sensors $s_{1}$ and $s_{4}$ need to be activated. Taking all targets into consideration simultaneously, this problem becomes more challenging.

To solve our problem, we map the minimum *ϵ*-detection coverage problem into a set select problem by constructing the candidate coverage set. We then propose an efficient approximation algorithm, Probabilistic Sensor Coverage Algorithm (PSCA), with a provable bound. Our main contributions are summarized as follows:

1. By theoretical analysis, we determine the detection boundary $d_{max}$ for the minimum *ϵ*-detection coverage problem with omni-directional probabilistic sensors.
2. By reducing a unit disk cover problem to the minimum *ϵ*-detection coverage problem, we prove it is NP-hard and map it into a set select problem, which can be solved by a set select algorithm.
3. We propose a bounded approximation algorithm, the PSCA, to solve the minimum *ϵ*-detection coverage problem and present the theoretical analysis of the algorithm approximation ratio.

The remainder of the paper is organized as follows: In Section 2, we summarize the related work, followed by the formulation of the minimum *ϵ*-connected coverage problem in Section 3. Section 4 provides the theoretical analysis of the problem. Section 5 presents our algorithm design and analysis. Simulation results are presented in Section 6, and finally, we conclude the paper in Section 7.

## 2. Related Works

Coverage is a fundamental problem in wireless sensor networks. The coverage problem can be classified into three types [5,6]: target coverage (e.g., [7,8,9,10,11,12,13,14,15,16,17,18]), area coverage (e.g., [19,20,21,22,23,24,25,26,27,28]) and barrier coverage (e.g., [29,30,31,32,33,34]).

In [37], Wan et al. conducted a comprehensive study on target coverage systematically. They investigated the minimum wireless coverage, cheapest wireless coverage and max-life wireless coverage problems. They presented a Polynomial Time Approximation Scheme (PTAS) for MWCand two randomized algorithms for CWCand MLWC, respectively. In [18], Cai et al. proposed several algorithms to address the Multiple Directional Cover Sets (MDCS) problem of organizing the directions of sensors into a group of non-disjoint cover sets for extending the network lifetime. Cardei et al. [16] studied the maximum lifetime problem of target coverage. The authors divided all sensors into disjoint sets, which can cover all targets. They proposed an efficient method to extend the sensor network life time by activating those sets successively. Since random deployment may not meet the coverage requirements of the target, Liao Zet al. [38] applied mobile sensors to the connected coverage problem. Their objective is to optimize the movement distance of all sensors, subject to the coverage and connectivity requirements. The author in [39] studied the optimal movement of mobile sensors from the perspective of game theory to track the mobile targets. The connectivity of sensor nodes with the sink is also considered in the target coverage problems. The connected coverage problem aims at activating sensor nodes to monitor a finite set of targets and maintaining the connectivity of active nodes with the sink at the same time. In [40], Li et al. made a comprehensive survey of connected coverage. The authors firstly made a brief introduction to the graph models, such as random graphs and random geometric graphs. The optimal placement of sensor nodes was discussed next, which has a fundamental impact on the connectivity and other operational requirements of WSNs. Han Ket al. studied the minimum connected coverage problem in [15]. The problem is minimizing the total energy cost of both sensing and connectivity. Considering different combinations of omni-directional and directional features in the sensor and antenna, they studied four cases. They proved that the minimum connected coverage problem is NP-hard under all of these cases and proposed approximation algorithms based on the Steiner tree algorithm.

The above research works are mainly focused on the target coverage problem based on the 0/1 disk model, which is only a coarse approximation to the practical sensing model. The probabilistic sensing model appears to be a more realistic sensing model [5]. Researchers have proposed several empirical formulas, such as the exponential attenuation probabilistic model [17,41]. Hefeeda et al. proposed an exponential attenuation probabilistic model in [42]. They assumed that the detection probability is a continuously-decreasing function of sensing distance. This model is applied in the coverage problem, and the probabilistic coverage algorithm is designed to evaluate the degree of confidence in detection probability. The probabilistic sensors are exploited to improve barrier coverage. Li et al. in [32] adopted the probabilistic sensing model proposed by Hefeeda [42] in the barrier coverage problem. They analyzed the detection probability of arbitrary intrusions and address the problem of scheduling sensors to guarantee *ϵ*-barrier coverage with energy efficiency. The author formulated a minimum weight *ϵ*-barrier problem, which aimed to schedule sensor energy usage efficiently. Based on the Voronoi graph and maximum flow, they proposed a bounded approximation algorithm to schedule the activation of sensors.

The paper [43] discussed the target coverage based on probabilistic sensors. The problem is minimizing the costs of a network deployed for target coverage. They formulated the problem as an optimization problem. A genetic algorithm is used to optimize the total costs of the network. Dimitrios et al. [44] focused on prolonging network lifetime under probabilistic target coverage. The author defined the minimum detection accuracy problem, where the objective was to prolong the network lifetime as much as possible and to achieve a minimum allowed detection probability for all of the targets in the network. A localized algorithm, LoCQAl, was designed to solve the previous problem.

In [45], both target coverage and connectivity were considered. They solved the Connected Target Coverage (CTC) problem under the probabilistic model. Assuming that each target was detected beyond the predefined threshold by at least one sensor, they reduced the CTC problem under the probabilistic model to the CTC problem under the 0/1 disk model. The directional probabilistic sensor was adopt in [46]. The author focused on the k-coverage problem, where the object was minimizing the number of active sensors. The evaluation shows that the two algorithms proposed by the author outperform the approach of [1].

In summary, [32] concentrates on the barrier coverage, while [43,44,45] belong to target coverage. The approach proposed by [32,44,45] is not applicable for us, since their work is designed for maximum lifetime, while we take account of minimum energy in this paper. The author in [43] has proposed an algorithm based on the genetic algorithm to minimize the costs of a network deployed for target coverage. The realistic case studies demonstrate very promising results.

## 3. Preliminary and Problem Formulation

In this study, we focus on the target detection coverage problem based on probabilistic sensors; that is, how to deploy sensors properly to meet the requirements of the target detection coverage. First, we describe the sensing model in detail and introduce the target detection deployment scenarios based on the probabilistic sensors. Then, we define the minimum *ϵ*-detection coverage problem based on the probabilistic sensing model. For the targets to be monitored in an area, our aim is to deploy the least probabilistic sensors that enable the detection probability of each target to be no less than *ϵ*.

### 3.1. Sensing Model

Sensors monitor the targets by receiving the energy emitted by targets. The energy received by sensors is attenuated, and the detection probability is reduced as the distances between the targets and the sensor nodes increase.

**Figure 2:**
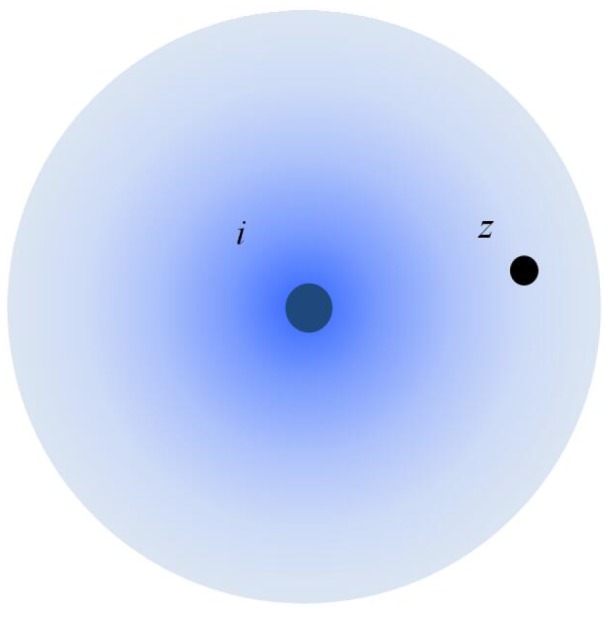
Omni-directional probabilistic sensor.

**Figure 3:**
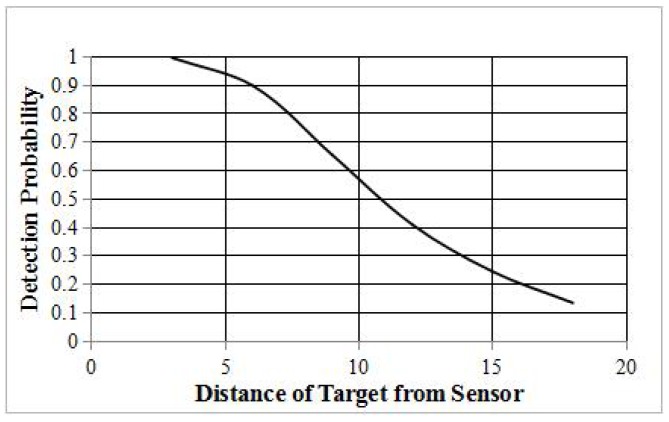
Change in detection probability with distance (m).

In this study, we take the omni-directional probabilistic sensors into consideration. As shown in Figure 2, an omni-directional probabilistic sensor can detect the state in all 360 of the surrounding environment. The probability of detecting targets is a continuously-decreasing function [5] of the distance between sensors and targets, p=λ(d), where *d* is the distance between sensors and the targets and p=λ(∗) is a continuously-decreasing distance-dependent attenuation function demonstrating the sensing characteristics. Several empirical formulas have been proposed (e.g., [17,42,47]). Assuming that the energy emitted by targets is the same, Figure 3 describes the relationship between detection probability and distance in the exponential attenuation probabilistic model proposed by [42]. An important application of sensor networks is to detect some events occurring at some location. In our study, a target represents a static object that generates some event signals periodically, such as the acoustic signal. Multiple sensors will detect the occurrence of the event by receiving the signal from a target object. Due to the uncertainty of probabilistic sensors, the event signals will be captured by sensors in a certain probability. We aim to detect those events with the least probability *ϵ*, instead of 100%. If the object generates *n* events, the expectation of the captured events is n×ϵ.

Target: In our study, a targets *t* represent a target object that generates events randomly. The sensors deployed capture events generated by the target object by receiving the signal from the target object. To detect each event beyond *ϵ* means to detect the target object beyond *ϵ*.

For omni-directional probabilistic sensors, we define the detection probability of a target *z* detected by a sensor *i*: $p_{i}\left(z\right)=\lambda \left(dis\left(i,z\right)\right)$. The detection probability is the same in each concentric circle, while it decreases with the distance increment around the sensor location.

### 3.2. Network Model

We deploy *N* probabilistic sensors randomly in an *L* × *L* 2D plane. Let *V* denote the set of sensor nodes. In the same area, a set of targets *D*:D∩V = ø to be monitored by the sensor nodes in *V*. Each target can be monitored by multiple sensors nearby. The location of sensors and targets can be obtained by prior localization methods. There exists a sink node. All active sensors must be connected to the sink (probably though some relay nodes).

Due to the uncertainty of probabilistic sensors, the overhead of the network will be enlarged. In our study, we assume that the energy cost of a sensor contains two factors: sensing and communication. The overhead of whole networks equals the sum of the sensors’ energy. The work in [15] shows that the communication cost dominates the total energy consumption in a sensor node. Thus, the increment of overhead in coverage is not significant. To narrow down the overhead of networks, we should activate less relay sensors.

### 3.3. Problem Statement

The target coverage problem requires that the detection probability of each target is not less than the detection probability threshold *ϵ* by activating some nodes from the randomly-deployed sensor nodes. Based on the probabilistic sensing model described in the Section 3.1, we give the definition of detection probability and a formal description of the problem.

For each target z∈D, $p_{i}\left(z\right)$ denotes the detection probability of *z* monitored by sensor *i*.

Assuming that $s_{z}$ is the sensor set around *z*, the detection probability of a target *z* is P(z). P(z) is computed by the probability formula, which integrates the detection probability of each sensor in $s_{z}$, i.e.,

(1) $$P\left(z\right)=1-\prod_{i\in s_{z}}\left(1-p_{i}\left(z\right)\right)$$

Detection probability threshold *ϵ*: *ϵ* is a threshold set by applications, such that the detection probability of each target detected by the active sensors is at least *ϵ*.

Associated with the detection probability threshold *ϵ*, we present the definition of the minimum *ϵ*-detection coverage problem based on omni-directional probabilistic sensors.

Minimum *ϵ*-detection coverage problem: Given a set of sensors *V* = {1,2,…,N}, and the sensors are randomly deployed in an *L* × *L* 2D Euclidean plane. The targets set is *D*. *m* targets are arbitrarily distributed in the 2D plane. We aim to activate a subset of sensors C⊆V such that the detection probabilities for all targets are not less than the threshold *ϵ*. If there is more than one set that satisfies the detection requirement, then the set with the minimum number of sensors is the minimum *ϵ*-detection coverage set.

### 3.4. Connectivity

To solve the minimum *ϵ*-detection coverage problem, a set of sensors will be activated. Each active sensor that needs to be connected to the sink (probably though relay nodes) has a transmission radius $R_{t}$. We assume that they can communicate with each other if their Euclidean distance is no more than $R_{t}$. We name it the relay sensor if it has been just activated for communication.

Connected graph: We use G=({s}∪V,E) to denote the connected graph, where *s* is the sink and *E* is the edge set. For $\forall v^{\prime }\in V$, $\forall v^{\prime \prime }\in V$, if $dis\left(v^{\prime },v^{\prime \prime }\right)<R_{t}$, an undirected edge $\left(v^{\prime },v^{\prime \prime }\right)$ will be added into *E*. For $\forall v^{\prime }\in V$, if $dis\left(v^{\prime },s\right)<R_{t}$, we connect them by an undirected edge $\left(v^{\prime },s\right)$.

To provide connectivity, we use a two-approximation Steiner tree algorithm [48] to find a Steiner tree $T_{s}$ that spans the sink and all active sensors in connected graph *G*. The Steiner points in $T_{s}$ will be activated as relay sensors.

## 4. Problem Analysis

We present the definition of detection gain in this section. We can easily determine whether a target surpasses *ϵ* by computing the detection gain. We will prove that the minimum *ϵ*-detection coverage problem is NP-hard via a special case.

### 4.1. Analysis of the Detection Probability

Given a target *z* detected by a sensors set $s_{z}$, the detection probability is $P\left(z\right)=1-\prod_{i\in s_{z}}\left(1-p_{i}\left(z\right)\right)$.

If P(z) is larger than *ϵ*, then we can get:

(2) $$P\left(z\right)=1-\prod_{i\in s_{z}}\left(1-p_{i}\left(z\right)\right)\geq \epsilon$$

We linearize the formula as follows:

(3) $$\begin{array}{c} P\left(z\right)=1-\prod_{i\in s_{z}}\left(1-p_{i}\left(z\right)\right)\geq \epsilon \\ \Rightarrow 1-\epsilon \geq \prod_{i\in s_{z}}\left(1-p_{i}\left(z\right)\right)\\ \Rightarrow \ln\left(1-\epsilon \right)\geq \sum_{i\in s_{z}}\ln\left(1-p_{i}\left(z\right)\right)\\ \Rightarrow -\ln\left(1-\epsilon \right)\leq -\sum_{i\in s_{z}}\ln\left(1-p_{i}\left(z\right)\right) \end{array}$$

The term Ψ=−ln(1−ϵ) is defined as the aggregate gain threshold.

Sensor detection gain $\phi_{i}\left(z\right)$: A target can get detection gain from sensor *i*, and the formula is $\phi_{i}\left(z\right)=-\ln\left(1-p_{i}\left(z\right)\right)$.

The detection gain is used to estimate a sensor’s influence on some target. When $p_{i}\left(z\right)$ is less than a given threshold, we ignore the detection probability and detection gain, i.e., $p_{i}\left(z\right)=\phi_{i}\left(z\right)=0$. If the detection probability or detection gain is zero, we think the sensor could not monitor the target. Details on the given threshold are discussed in later sections.

Cumulative detection gain $\sum_{i\in s_{z}}\phi_{i}\left(z\right)$: We can get a target’s cumulative detection gain by aggregating detection gains from surrounding sensors.

Obviously, if target *z* satisfies the detection requirement, then the cumulative detection gain of *z* is bigger than Ψ, i.e., $\sum_{i\in s_{z}}\phi_{i}\left(z\right)\geq \Psi$.

### 4.2. 0–1 Integer Programming Problem

Let us consider a field consisting of *n* sensors and *m* targets. The sensors and targets are randomly distributed. $x_{i}$, i∈1,2,…,n is the decision variables. $x_{i}$ is one if the sensor *i* is activated, and zero otherwise. Let $z_{j}$, j∈1,2,…,m represent targets.

The minimum *ϵ*-detection coverage problem can be formulated as follows:

(4) $$\begin{array}{ll} & \;min\;\;x_{1}+x_{2}+\ldots +x_{n}\\ s.t. & \left\{\begin{array}{c} \sum_{i}^{n}\phi_{i}\left(z_{j}\right)\times x_{i}\geq \Psi ,j=1,2,\ldots ,m\\ x_{i}=0\;or\;x_{i}=1,i=1,2,\ldots ,n \end{array}\right. \end{array}$$

The optimization is to minimize the number of active sensors. Each sensor is in either the active state or the sleep state, and each target’s cumulative detection gain ought to be greater than the aggregate gain threshold.

### 4.3. NP-Hardness Proof

In this section, the minimum *ϵ*-detection coverage problem is proven to be NP-hard theoretically by proving the unit disk cover problem ≤*p* the minimum *ϵ*-detection coverage problem.

**Theorem** **1.** The minimum ϵ-detection coverage problem is NP-hard.

**Proof** **of** **Theorem** **1.** Unit disk cover problem: Given a set $P=\{e_{1},e_{2},\ldots ,e_{m}\}$ of *m* points and a set $A=\{A_{1},A_{2},\ldots ,A_{n}\}$ of *n* unit disks in the Euclidean plane, the objective is to select minimum cardinality subset $A^{*}\subseteq A$, such that each point in *P* is covered by at least one disk in $A^{*}$. It is an NP-complete problem [49].

According to the proven NP-hardness, for any instance in the unit disk cover problem, it should be reduced into minimum *ϵ*-detection coverage problem in polynomial time. Reducing the procedure will be presented in detail.

**Figure 4:**
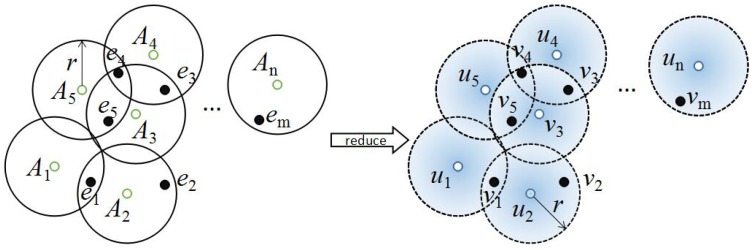
A reduction from the unit disk cover problem to the minimum *ϵ*-detection coverage problem. We set $r_{s_{k}}=r$ and $\epsilon =e^{-\beta r_{s_{k}}}$.

As shown in Figure 4, for each unit disk $A_{i}\in A$ with radius *r*, we create a probabilistic sensor $u_{i}$ in the corresponding position. For each $e_{i}\in P$, $e_{i}$ is denoted by a corresponding target $v_{i}$. For the deployed sensors, we take the probabilistic model in [43] as follow:

(5) $$p_{i}\left(z\right)=\left\{\begin{array}{cc} e^{-\beta dis(i,z)}, & if\;dis\left(i,z\right)\leq r_{s_{k}}\\ 0, & otherwise \end{array}\right.$$

To reduce the unit disk cover problem to our minimum *ϵ*-detection coverage problem, we set $r_{s_{k}}=r$ and $\epsilon =e^{-\beta r_{s_{k}}}$. This is the most critical part of the proof. Under these settings, the probability $p_{i}\left(z\right)$ satisfies:

(6) $$\begin{array}{c} \left\{\begin{array}{ll} p_{i}\left(z\right)\geq \epsilon =e^{-\beta r}, & if\;dis\left(i,z\right)\leq r\\ p_{i}\left(z\right)=0, & if\;dis\left(i,z\right)>r \end{array}\right. \end{array}$$

This means that the minimum detection probability between a sensor and a target is *ϵ*. The detection probability below *ϵ* will be regarded as zero. According to Equation (1) and the settings ($r_{s_{k}}=r$, $\epsilon =e^{-\beta r_{s_{k}}}$), to achieve the detection probability threshold *ϵ*, the probability of target *z* satisfies:

(7) $$\begin{array}{c} P\left(z\right)=1-\prod_{i\in s_{z}}\left(1-p_{i}\left(z\right)\right)\geq \epsilon =e^{-\beta r_{s_{k}}}\\ \Rightarrow 1-\epsilon \geq \prod_{i\in s_{z}}\left(1-p_{i}\left(z\right)\right) \end{array}$$

With the settings ($r_{s_{k}}=r$ and $\epsilon =e^{-\beta r_{s_{k}}}$) and Equation (6), there must be at least one sensor $i\in s_{z}$ such that probability $p_{i}\left(z\right)\geq \epsilon$ to satisfy Equation (7). In other words, if and only if there is at least one sensor far from the target *z* within distance *r*, the target *z* will be detected beyond *ϵ*.

Thus, in Figure 4, a target $v_{i}$ will be covered with detection probability beyond *ϵ*, if the target is located in the disk centered in the position of some sensor $u_{i}$ with radius *r*. The detection probability of some target $v_{j}$ is zero, while $v_{j}$ is outside all disks centered in the position of sensors $\left\{u_{1},u_{2},\ldots u_{n}\right\}$ with radius *r*. Under these settings ($r_{s_{k}}=r$ and $\epsilon =e^{-\beta r_{s_{k}}}$) of the probabilistic model, the minimum *ϵ*-detection coverage problem is to find the least disks with radius *r* centered in sensors $\left\{u_{1},u_{2},\ldots u_{n}\right\}$ to cover all targets, which is exactly the same as the unit disk cover problem. Therefore, any instance of the unit disk cover problem can be reduced to the instance of the minimum *ϵ*-detection coverage problem in polynomial time. Thus, the minimum *ϵ*-detection coverage problem must also be NP-hard. ☐

### 4.4. Problem Transformation

As the detection gain is small when the distance between a sensor and a target is farther, we assume that the sensor could not detect the target if the detection gain is too small, and we regard the detection gain as zero.

Minimum detection probability $p_{min}$: $p_{min}$ is a threshold set by applications. If the detection probability of a target *t* is detected by one sensor *s* to be less than $p_{min}$, we take it as zero, otherwise λ(dis(s,t)).

We use the minimum detection probability $p_{min}$ to determine the detection boundaries of sensors. For a given target, it can be detected with a certain probability by a sensor if its detection probability is larger than $p_{min}$. Therefore, we can get the effective detection radius of sensors: $d_{max}=\lambda^{-1}\left(p_{min}\right)$. This means that if the distance dis(s,t) between the target *t* and the sensor *s* is beyond $d_{max}$, the detection probability will be treated as zero. We compute the detection probability and the detection gains only when the distance between sensors and targets is less than $d_{max}$.

**Figure 5:**
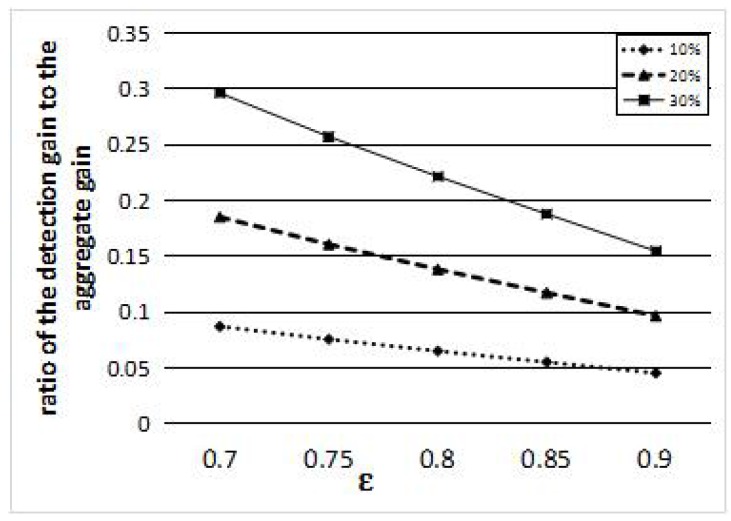
The ratio of the detection gain to the aggregate gain with different thresholds *ϵ*.

In Figure 5, we demonstrate the ratio of the detection gain to the aggregate gain threshold. The different curves in Figure 5 represent the different detection probabilities. The detection probability threshold *ϵ* is the horizontal axis, and the ratio is the vertical axis. For example, when the detection probability threshold *ϵ* is 0.7 and the detection probability is 10%, the ratio of the detection gain to aggregate gain threshold is 8.75%. In this case, we can set the $p_{min}$ 0.1, as for the detection gain with the detection probability less than 0.1 accounts for a small part of the aggregate gain threshold. The ratio can be used to demonstrate how much impact is generated by a sensor on a target. Therefore, how do we determine the $p_{min}$ with notable impact? We just set the $p_{min}$ such that the ratio of the detection gain to the aggregate gain threshold $\frac{-ln(1-p_{min})}{-ln(1-\epsilon )}$ is larger than a pre-defined ratio *τ*. Thus, the $p_{min}$ can be calculated by the following formula:

(8) $$p_{min}=1-{\left(1-\epsilon \right)}^{\tau }$$

Independent coverage set: A target’s independent coverage set is c(z)⊆V, satisfying $\sum_{i\in c(z)}\phi_{i}\left(z\right)\geq \Psi$ and $\forall c^{\prime }\left(z\right)⊊c\left(z\right)$, $\sum_{i\in c(z)}\phi_{i}\left(z\right)<\Psi$.

**Figure 6:**
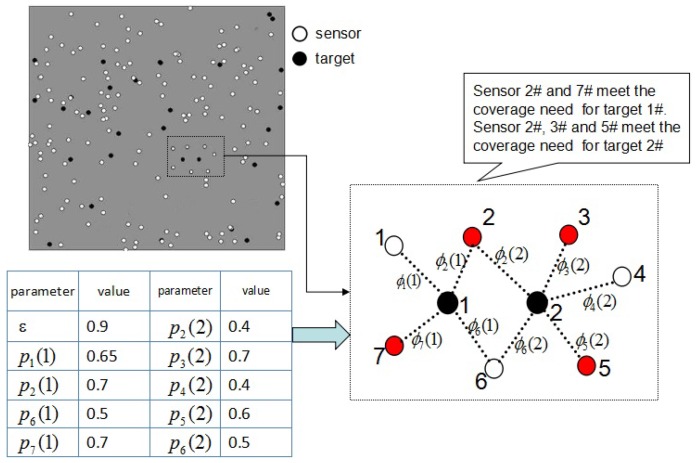
An example of the independent coverage set. The parameters are listed in the left table. $p_{i}\left(j\right)$ determines the probability of target *j* detected by the sensor *i*. The weight of the dotted line $\phi_{i}\left(j\right)$ determines the detection gain of target *j* from sensor *i*. The red point in the figure represents the active sensor.

The concept of independent coverage set is to make full use of as few sensors as possible to meet the detection requirements. A target obtains the cumulative detection gains from sensors in its independent coverage set, which exactly exceeds the aggregate gain threshold, and there are no redundant sensor nodes. Assuming the detection probability threshold *ϵ* 0.9, {2,7} is the independent coverage set of Target 1#and {2,3,5} of Target 2# in Figure 6.

Candidate coverage set: Target *z*’s candidate coverage set is the union of its independent coverage sets, $C\left(z\right)=\cup \{c_{i}\left(z\right)\},i\in \left\{1,2,\ldots ,k\right\}$. $c_{i}\left(z\right)$ is the *i*-th independent coverage set of *z*, and for any $c_{i}\left(z\right)$, $c_{i}\left(z\right)\in C\left(z\right)$.

Based the above analysis, we can redefine the minimum *ϵ*-detection coverage problem as follows: In an L×L 2D plane, *m* targets ought to be monitored. For every target *z*, we select an independent coverage set from its candidate coverage set. Our object is to minimize the number of sensors of the union of the *m* independent coverage sets.

We present an enumeration algorithm to finding the candidate coverage set of any target in Algorithm 1.
**Algorithm 1 CCS (Candidate Coverage Set Algorithm).****Input**: A target *z*, sensor set $s_{z}$ around *z*, aggregate gain threshold Ψ**Output**: Candidate coverage set C(z) of target *z*
1:C(z)= ∅2:c(z)= ∅3:*sort sensors in s_z_ by gains in descending order* 4:*call* **DFS**(0, 0, *c*(*z*))5:**return***C*(*z*)
**void DFS**(Index,totalGains)
1:**if** $Index\geq s_{z}.size$ **then**2:    **return**3:**end if**4:**if** totalGains≥Ψ **then**5:    C(z)=C(z)∪{c(z)}6:    **return**7:**end if**8:DFS(Index+1,totalGains,c(z))9:$sensor=s_{z}\left[Index\right]$10:$gains=\phi_{s}ensor\left(t\right)$11:c(z)=sensor∪c(z)12:DFS(Index+1,totalGains+gains)



## 5. Algorithm Design

### 5.1. PSCA Approximation Algorithm

According to the definition of the candidate coverage set, we design an enumeration algorithm (CCS (Candidate Coverage Set Algorithm)) to find the candidate coverage set of any target. As the minimum *ϵ*-detection coverage is an NP-hard problem, we design an approximation algorithm (PSCA (Probabilistic Sensor Coverage Algorithm)) based on the greedy strategy. We divide the algorithm into three main phases: the computation phase, the selection phase and the connection phase. In the computation phase, we call Algorithm 1 to calculate the candidate coverage set of each target in *D*. In the selection phase, we select an independent coverage set from every target’s candidate coverage set in turn. Because there are *m* targets in *D*, we will select *m* independent coverage sets in total. The union of the *m* independent coverage sets is the final result. The above selection phase is described in Algorithm 2. In the connection phase, we use a Steiner tree algorithm to select some sensors as relay nodes that maintain the network connectivity.
**Algorithm 2 PSCA (Probabilistic Sensor Coverage Algorithm).****Input**: Candidate coverage set $C_{i},\;i=1,2,\ldots ,m$, sensor set V={1,2,…,n}**Output**: *S*
1:S= ∅2:U={1,2,…,m}3:**for** ifrom1tom **do**4:    F[i]=05:    **for** jfrom1tondo6:        **if** $sensor\;j\;exists\;in\;C_{i}\;$**then**7:           F[j]=F[j]+18:        **end if**9:    **end for**10:**end for**11:**for** ifrom1tom **do**12:    select an independent coverage set *c* from a candidate coverage set $C_{k}$, k∈U that S∪c is minimum, if multiply *c* satisfy the needs, choose the *c* that has the biggest weight.13:    U=U−{k}14:    S=S∪c15:**end for**16:Call a Steiner tree algorithm [48] to find a Steiner tree $T_{S}$ that spans the sensors in {s}∪S. Activate the Steiner points in $T_{S}$ as relay sensors.17:**return** S



As an example, in Figure 6, we will demonstrate the PSCA in detail. There are two targets and seven sensors in the region. The parameters are shown in Figure 6. All targets ought to be detected by the active sensors by at least 0.9. The candidate coverage sets of all targets calculated by Algorithm 1 are presented in Table 1. As shown in Table 1, Target 1 has three independent coverage sets, and the composition of the three sets is the candidate coverage set of Target 1. Target 2 has five independent coverage sets.

Frequency of sensor iF[i]: We use F[i] to denote that the frequency of sensor *i* occurs in the candidate coverage sets of all targets.

Firstly, PSCA will count the frequency of every sensor. For example, the frequency of Sensor 2 is two, because it occurs in the candidate coverage sets of Target 1 and Target 2. The frequency of Sensor 7 is one, as for it occurs in the candidate coverage set of Target 1.

Weight of the independent coverage set: The weight of an independent coverage set is the sum of the frequency of every sensor in the independent coverage set; as the independent coverage set {2,7}, the weight is F[2]+F[7]=3.

PSCA will select an independent coverage set from the candidate coverage sets of uncovered targets in each selection. Once an independent coverage set of target *z* is chosen, the target *z* will be marked as covered by activating sensors in the independent coverage set. Additionally, the sensors in the independent coverage set will be pushed into *S*. PSCA will select the independent coverage set *m* times, for the number of targets is *m*. In each selection, the strategy is to choose the independent coverage set such that the union of the independent coverage set and *S* has the least sensors. If more than one independent coverage sets meet the above standard, we choose the one with the highest weight.

**Figure 7:**
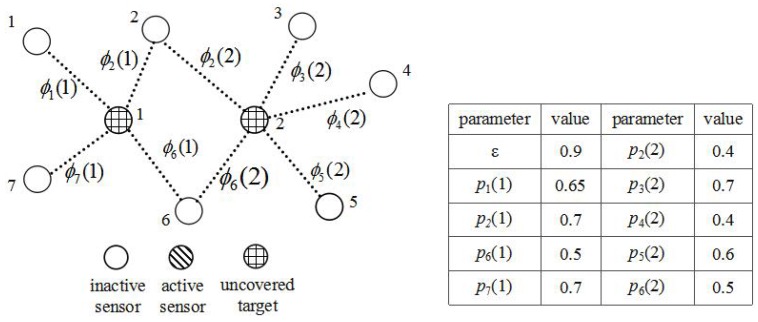
The initial state of WSNs. No sensors are activated, and no targets are covered.

In initial stage (from Line 1 to Line 10), we set *S* empty, which is used to store the active sensors. The set *U* is initialized {1,2,…,m}, which is used to store the uncovered targets. Each sensor’s frequency will be counted. Details about the initial state of PSCA are shown in Table 2. The initial state of the sensor network is shown in Figure 7. No sensors are activated, and no targets are covered.

**Figure 8:**
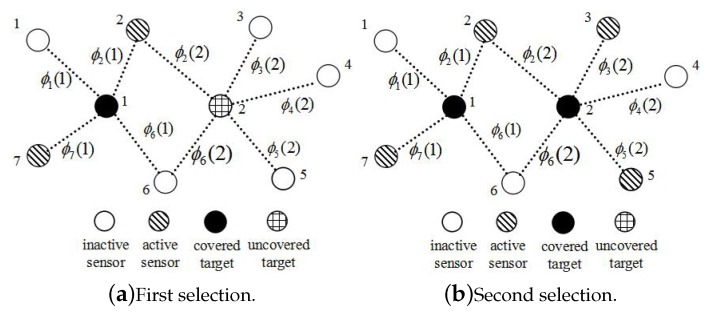
In the first selection, Target 1 is covered. Sensor 2 and Sensor 7 are activated. In the second selection. All targets have been covered by four sensors with the least detection probability threshold *ϵ*.

In the selection stage (from Line 11 to 15), PSCA will select two independent coverage sets, as there are two targets in the sensor network. In Table 3a, PSCA first selects the independent coverage set {2,7}, because the number of sensors in {2,7}∪S is minimum. Then, the sensors in {2,7} will be activated and pushed into *S*. Target 1 will be marked covered, and we pop it out from *U*. Details about the state of the sensor network are demonstrated in Figure 8a.

Next, PSCA will select the independent coverage set of targets in *U*. Because the union of *S* and the independent coverage set {2,3,5} and the union of *S* and {2,3,6} have the least sensors, the weight of {2,3,5} and {2,3,6} will be calculated. The weight of {2,3,5} is four, and so is {2,3,6}. PSCA will randomly choose the one with the biggest weight. As shown in Table 3b, PSCA selects {2,3,5}. The sensors in {2,3,5} will be activated and pushed into *S*. Target 2 will be marked covered, and we pop it out of *U*. We show the state of the sensor network after this selection in Figure 8b.

Now, the *U* becomes ∅, which indicates that all targets will be covered by sensors in *S* at least *ϵ*. The *S* is the ultimate result, and our algorithm returns *S*.

### 5.2. Algorithm Theoretical Analysis

The approximation bound of our algorithm is discussed theoretically in this section.

Let the size of *S* calculated by PSCA be *N* and the size of optimum $S^{*}$ be $N_{opt}$.

**Theorem** **2.** $\frac{N}{N_{opt}}\leq m\frac{\ln(1-\lambda \left(dis_{min}\right))}{1-p_{min}}$

**Proof** **of** **Theorem** **2.** PSCA initializes the *S* = ∅, and it will select independent coverage sets *m* times. We define $∆N_{i}$ as the number of sensors in *S* increases, and $k_{i}$ as the target whose candidate coverage set PSCA chooses from at the *i*-th step. Then, $N=∆N_{1}+∆N_{2}+\ldots +∆N_{m}$. Let $S_{i}$ be the result of *S* after the *i*-th step. Specifically, $S_{0}$ is ∅.

Due to the independent coverage set, we choose to make the increment of *S* minimum; obviously, we get:

(9) $$\begin{array}{c} ∆N_{1}=min_{c_{i}\in C_{k_{1}}}\left|c_{i}-S_{0}\right|=min_{c_{i}\in C_{k_{1}}}\left|c_{i}\right|\\ ∆N_{2}=min_{c_{i}\in C_{k_{2}}}\left|c_{i}-S_{1}\right|\leq min_{c_{i}\in C_{k_{2}}}\left|c_{i}\right|\\ \ldots \\ ∆N_{m}=min_{c_{i}\in C_{k_{m}}}\left|c_{i}-S_{m-1}\right|\leq min_{c_{i}\in C_{k_{m}}}\left|c_{i}\right| \end{array}$$

Then, we have the following formula by accumulating the above inequality.

(10) $$N=∆N_{1}+∆N_{2}+\ldots +∆N_{m}\leq min_{c_{i}\in C_{k_{1}}}\left|c_{i}\right|+min_{c_{i}\in C_{k_{2}}}\left|c_{i}\right|+\ldots +min_{c_{i}\in C_{k_{m}}}\left|c_{i}\right|$$

For any j={1,2,…,m}, we have:

(11) $$min_{c_{i}\in C_{k_{j}}}\left|c_{i}\right|\leq \frac{\Psi }{\phi_{min}}=\frac{\Psi }{-\ln(1-p_{min})}$$

When the detection gains are the minimum $\phi_{min}$, the independent coverage set needs the most sensors. Let $\phi_{min}=-\ln\left(1-p_{min}\right)$ denote the minimum detection gains, where $p_{min}$ is the detection probability threshold. Thus, the size is less than or equal to $\frac{\Psi }{\phi_{min}}$ for any independent coverage set.

With Equations (10) and (11):

(12) $$N\leq \frac{m\Psi }{-\ln(1-p_{min})}$$

Since the size of $S^{*}$ $N_{opt}$ satisfies:

(13) $$N_{opt}\geq \frac{\Psi }{\phi_{max}}\geq \frac{\Psi }{-\ln(1-\lambda \left(dis_{min}\right))}$$

where $\phi_{max}$ is the maximum detection gains and $dis_{min}$ is the minimum distance between targets and sensors in WSNs.

Combing Equations (12) and (13), we get:

(14) $$\frac{N}{N_{opt}}\leq m\frac{\ln(1-\lambda \left(dis_{min}\right))}{\ln(1-p_{min})}$$

☐

Based on the analysis of the above sections, PSCA essentially solves a minimum union problem. For a given sensor set $V=\{s_{1},s_{2},\ldots s_{n}\}$, candidate sets $C_{1},C_{2},\ldots ,C_{m}$, we choose an independent coverage $c_{i}\subseteq V,i=1,2,\ldots ,m$ from every candidate sets *m* times. We aim to get the minimum union $\cup_{i=1}^{m}c_{i}$.

The minimum union problem and the maximum intersection problem are equivalent based on the next equation:

(15) $$min\left|\overset{m}{\underset{i=1}{⋃}}c_{i}\right|=min\left|\bar{\overset{m}{\underset{i=1}{⋂}}\bar{c_{i}}}\right|=|V|-max\left|\overset{m}{\underset{i=1}{⋂}}\bar{c_{i}}\right|$$

For the maximum intersection problem, Clifford et al. [50] has proven that the problem cannot be approximated within an $n^{1-\epsilon }$ multiplicative factor, for any ϵ>0, unless NP=P.

**Theorem** **3.** *The average time complexity of Algorithm 1 is* $O(2^{\frac{n\pi d_{max}^{2}}{L^{2}}})$*, and the time complexity of PSCA is* $O(m^{2}nk)$ *(k is the maximum number of independent coverage set in candidate coverage set).*

**Proof** **of** **Theorem** **3.** Due to Algorithm 1 being an enumeration algorithm based on DFS, Algorithm 1 is an exponential complexity algorithm. The exponential of Algorithm 1 is related to the number of sensors in $s_{z}$. As Section 4.4 mentioned, we take the probabilities into consideration only when the distance between sensors and targets is less than $d_{max}$. As for the targets and sensors being uniformly distributed in an L×L area, the average number of sensors in $s_{z}$ equals $\frac{n\pi d_{max}^{2}}{L^{2}}$. Thus, the average time complexity of Algorithm 1 is $O(2^{\frac{n\pi d_{max}^{2}}{L^{2}}})$.

In Line 6 of PSCA, if we want to know whether sensor *j* exists in $C_{i}$, we need to visit every independent coverage set in $C_{i}$. Thus, it costs O(k) time. From Line 3 to Line 10, the time complexity is O(mnk). In Line 12, we also need to visit every independent coverage set in the candidate coverage sets of uncovered targets. The union operation S∪c costs O(|S|)×O(|c|) (|S| is the number of sensor in *S*, so does |c|) time. In addition, |c| is at most $\frac{-ln(1-\epsilon )}{-ln(1-p_{min})}$, for the detection gain is at least $-ln(1-p_{min})$. Often, O(|c|) is less than a constant 20, and O(|S|) is obviously less than *n*. Therefore, PSCA costs O(m)×O(k)×O(|S|)×O(|c|)=O(mnk) time in Line 12. As mentioned above, we need to choose independent coverage sets *m* times. Therefore, PSCA costs $O\left(mnk\right)+m\times O\left(mnk\right)=O\left(m^{2}nk\right)$ time. In addition, the space complexity of PSCA depends on the size of candidate coverage sets, which is O(mk). ☐

## 6. Performance Evaluation

We conduct a series of simulations in order to evaluate the performance of PSCA. Sensors are randomly deployed in an L×L two-dimensional plane, with the constraint that every target’s detection probability can achieve the threshold *ϵ*. This study adopts the exponential attenuation probabilistic model proposed in [42].

The main objective of PSCA is to select the minimum number of sensors from the sensor set, and the selected sensors satisfy the minimum *ϵ* coverage requirements of each target. We conduct simulations in three different size areas and randomly deploy different numbers of sensors. Targets are randomly distributed in every area. We repeat the experiments 20 times for different detection probability threshold *ϵ* and different number of targets *m*. The communication cost of a sensor is set to two Joules. For simplicity, we assume each sensor has the same sensing cost of one Joule. For the relay sensor, the sensing cost is zero, since it is used for communication only.

### 6.1. PSCA’S Performance Evaluation

**Figure 9:**
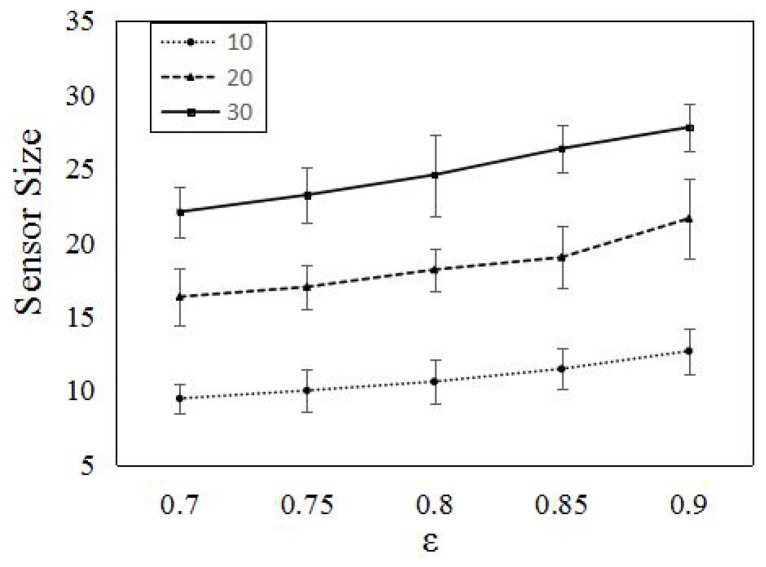
Comparing the numbers of active sensors for different target sizes and detection probability threshold in a 100 m × 100 m area. We randomly deployed 120 sensors in the area and set the minimum detection probability $p_{min}=0.2$. The numbers of targets are 10, 20, 30.

In this section, we conduct extensive simulations to evaluate the performance of PSCA. In Figure 9, we randomly deploy 120 omni-directional probabilistic sensors in the 100 m × 100 m area. We set the minimum detection probability $p_{min}=$ 0.2; the effective detection radius of the sensors is $\lambda^{-1}\left(p_{min}\right)\approx$ 16.5 m. First, we compare the numbers of sensors with different *ϵ*. As shown in Figure 9, with *ϵ* increasing, more sensors are needed. We also compare the number of sensors with different targets. Three curves in Figure 9 respectively represent that target Numbers 10, 20 and 30 required different numbers of sensors, respectively. The average number of sensors is less, while the target number is increasing. The plots suggest that when the target number is increasing, sensors will work more cooperatively.

**Figure 10:**
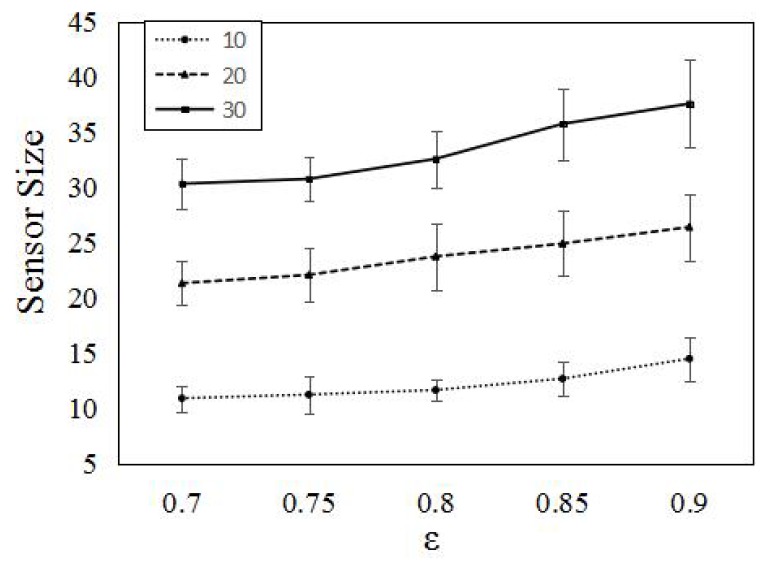
Comparing numbers of active sensors for different target sizes and coverage requirements in a 200 m × 200 m area. We randomly deployed 400 sensors in the area and set the minimum detection probability $p_{min}=0.2$. The numbers of targets are 10, 20 and 30.

We also do the simulation in a sparse area. The area in Figure 10 is 200 m × 200 m. We randomly deploy 400 omni-directional sensors with $p_{min}=0.2$. The $d_{max}$ is $\lambda^{-1}\left(p_{min}\right)\approx$ 16.5 m. The numbers of targets are set to 10, 20 and 30, respectively. Compared to Figure 9, the sensors in Figure 10 are sparser. We also conduct a series of simulations for different *ϵ*. From Figure 10, we clearly see that, for the same coverage requirement and target number, the larger the area is, the more sensors are activated.

### 6.2. Comparison Study

**Figure 11:**
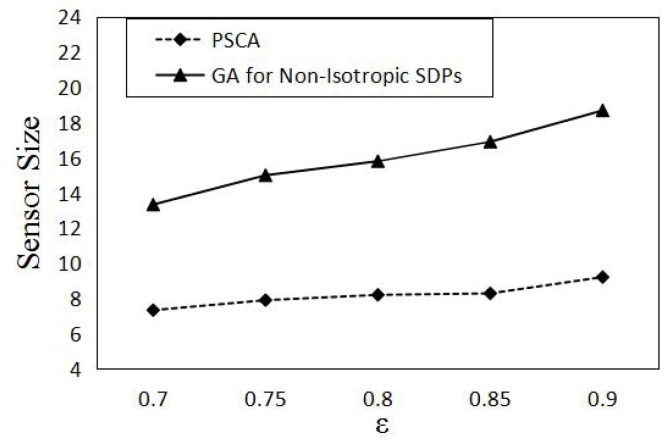
Sensor size vs. *ϵ*. Forty sensors are uniformly deployed in a 50 m × 50 m area. The number of targets is 10, and we set $p_{min}=0.2$.

**Figure 12:**
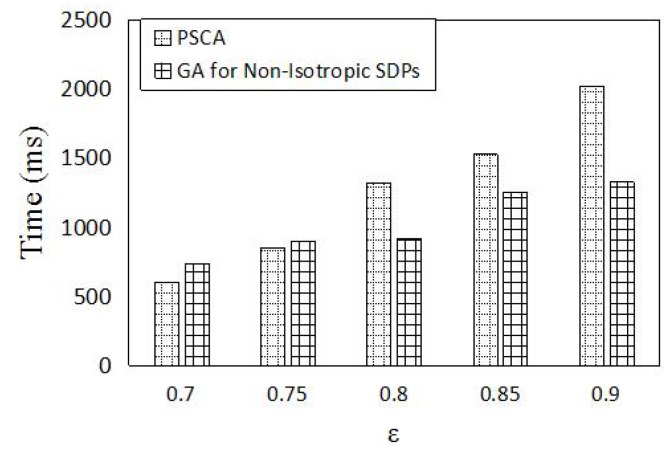
Computation time vs. *ϵ*. Forty sensors are uniformly deployed in a 50 m × 50 m area. The number of targets is 10, and we set $p_{min}=0.2$.

In this section, we compare the performance of PSCA against the genetic algorithm (GA) for non-isotropic sensor deployment problems (SDPs) [43] and Localized Coverage Quality Algorithm (LoCQAL) [44], in terms of performance. GA for non-isotropic SDPs is a genetic algorithm-based approach to solve the probabilistic target coverage problem. Figure 11 demonstrates the simulation result of the average number of sensors activated, while Figure 12 shows the computation time.

In the simulation experiment, we use the same sensing model for PSCA and GA for non-isotropic SDPs. Forty sensors are deployed randomly following a uniform distribution in a region of 50×50 m^2^. There are 10 targets randomly distributed in the same region. We set the minimum detection probability $p_{min}=0.2$. The parameters used for GA for non-isotropic SDPs are $TOURS=100,\;POP=50,p_{c}=0.9$ and $p_{m}=0.1$. As GA for non-isotropic SDPs aims to get the minimum cost of sensors, to apply it here, we set the cost of each sensor to one. Thus, we take the following fitness function for GA for non-isotropic SDPs in the simulation:

(16) $$f\left(x\right)=\sum_{j=1}^{n}x_{i}+w\left(m-coverage\right)$$

The meanings of $x_{i}$, *w* and coverage can be found in [43].

Figure 11 presents the average number of sensors activated by PSCA and GA for non-isotropic SDPs for different *ϵ*. As shown in Figure 11, PSCA activates a much lesser number of sensors than GA for non-isotropic SDPs. Since PSCA is based on a greedy search policy, in each selection of the independent coverage set, it always chooses the candidate set making the least increase for the cover set. While GA for non-isotropic SDPs is sensitive to the initial population of potential solutions, PSCA outperforms GA for non-isotropic SDPs.

We also compare the computation time versus different *ϵ* in Figure 12. Figure 12 shows that, when *ϵ* is less than 0.8, our algorithm outperforms GA for non-isotropic SDPs. This is because the number of independent coverage sets in candidate coverage sets of each target is not too large. The chosen stage of the independent set will not consume too much time. However, when *ϵ* becomes large, the combination of the independent coverage set will increase greatly. Much more computation time will be used for the chosen stage of the independent set. Thus, PSCA outperforms in computation time with a small *ϵ*.

**Figure 13:**
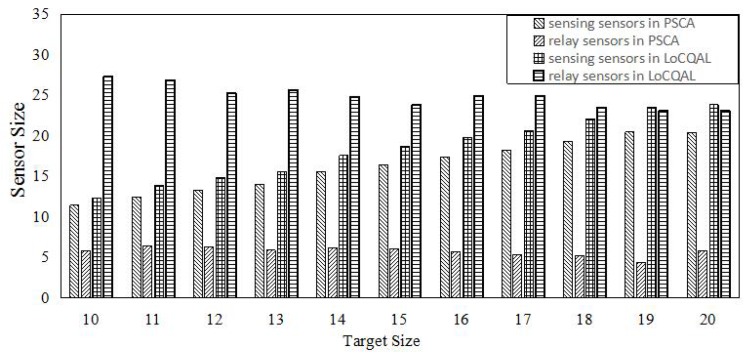
Impact of target size on PSCA and LoCQAL. One hundred and fifty sensors are deployed in a region of 150×150 m^2^. We fix *ϵ* to 0.7 and $p_{min}$ to 0.2.

In Figure 13, we demonstrate the performance comparison of PSCA and LoCQAL in terms of active sensors. To apply LoCQAL, we assume all sensors are static and that the sensors are sufficient to detect all targets beyond *ϵ* without movement. All active sensors can be divided into two categories: sensing sensor (detect targets) and relay sensor (just for information transmission). As shown in Figure 13, while the targets increase, we observe that LoCQAL has more relay sensors. This is because in LoCQAL, the sensors in the connected domination set are always activated. Moreover, PSCA outperforms LoCQAL in terms of sensing sensors.

**Figure 14:**
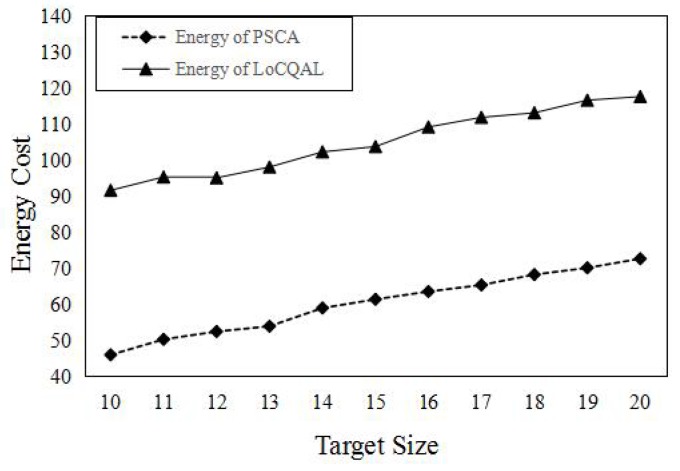
Energy cost comparison. For each sensor, the sensing cost is set to one Joule, and communication cost is two Joules.

The energy cost of a sensor contains two factors: sensing and communication. We denote the communication cost of a sensor as two Joules. We assume each sensor has the same sensing cost of one Joule. For the relay sensor, the sensing cost is zero, since it is used for communication only. In Figure 14, we compare PSCA with LoCQAL in terms of the total energy cost versus different target sizes. As for many redundant relay sensors in the connected domination set, this results in a huge energy waste. However in PSCA, sensors will be activated only when they are used to detect a target or they operate as relay nodes towards the sink.

## 7. Conclusions

In this paper, we studied the minimum *ϵ*-detection coverage problem. It aims at efficiently monitoring a finite set of targets using probabilistic sensors. We adopted omni-directional probabilistic sensors with the exponential attenuation probabilistic model. Based on the theoretical analysis of the probabilistic sensing model, we proposed the minimum *ϵ*-detection coverage problem and prove that it is NP-hard. We transformed the minimum *ϵ*-detection coverage problem into a minimum cover set selection problem, which is easily solved in a randomly-deployed sensor network. Then, we proposed the PSCA algorithm to solve the minimum *ϵ*-detection coverage problem. In addition, we presented the theoretical analysis of the algorithm approximation ratio and proved that PSCA has a bounded approximation. Finally, we performed extensive simulations and demonstrated the effectiveness of our algorithms. This paper focused on the investigation of the omni-directional probabilistic coverage model. The evaluation of our algorithm in the real test-bed is also a meaningful work in the future.

## Figures and Tables

**Table 1:** Candidate coverage sets.

Target	Candidate Coverage Set
1	{{1, 2, 6}, {2, 7}, {1, 6, 7}}
2	{{3, 4, 5}, {2, 3, 5}, {2, 3, 6}, {2, 4, 5, 6}, {3, 4, 6}}

**Table 2:** *F*[1] = 1, *F*[2] = 2, *F*[3] = 1, *F*[4] = 1, *F*[5] = 1, *F*[6] = 2, *F*[7] = 1.

Target	Candidate Coverage Set	S	U
1	{{1, 2, 6}, {2, 7}, {1, 6, 7}}	∅	{1, 2}
2	{{3, 4, 5},{2, 3, 5},{2, 3,6}, {2, 4, 5, 6}, {3, 4, 6}}		

**Table:** (a) First selection.

Target	Candidate Coverage Set	S	U
1	{{1, 2, 6}, {2, 7}√, {1, 6, 7}}	{2, 7}	{2}
2	{{3, 4, 5}, {2, 3, 5}, {2, 3, 6}, {2, 4, 5, 6}, {3, 4, 6}}		

**Table:** (b) Second selection.

Target	Candidate Coverage Set	S	U
1	{{1, 2, 6}, {2, 7}√, {1, 6, 7}}	{2, 3, 5, 7}	∅
2	{{3, 4, 5}, {2, 3, 5}√, {2, 3, 6}, {2, 4, 5, 6},{3, 4, 6}}		
